# Prevalence of gambling behaviours and their associations with socioemotional harm among 11–16 year olds in Wales: findings from the School Health Research Network survey

**DOI:** 10.1093/eurpub/ckz176

**Published:** 2019-10-03

**Authors:** G J Melendez-Torres, Rebecca E Anthony, Gillian Hewitt, Simon Murphy, Graham F Moore

**Affiliations:** Centre for the Development and Evaluation of Complex Interventions for Public Health Improvement, School of Social Sciences, Cardiff University, Cardiff, UK

## Abstract

**Background:**

Gambling opportunities are increasingly available and acceptable to many adolescents. Adolescent problem gambling has been associated with poor outcomes, such as lower reported physical and mental health. While much research has focussed on ‘problem’ gambling, analysing the distribution and determinants of experimentation with gambling is important in order to understand its normalization and population level consequences. This study describes the distribution of inequalities and socioemotional harms associated with adolescent gambling.

**Methods:**

Data were drawn from a subsample of students (*N* = 37 363) who completed gambling questions as part of the 2017 School Health Research Network Student Health and Wellbeing Survey, representing 193 secondary schools in Wales. Using imputations, we estimated a series of single-predictor and multi-predictor regressions for count of gambling behaviours, any gambling in the past 12 months and socioemotional harms of gambling.

**Results:**

Approximately two-fifths (41.0%) of respondents reported gambling in the past 12 months, of whom 16.2% reported feeling bad as a result of their own gambling. We found significant sex differences in gambling, with boys gambling more frequently than girls. Adolescents from more affluent families reported a higher count of gambling behaviours and socioemotional harms, although paradoxically, increasing affluence was also associated with lower prevalence of gambling in the last year. Non-White British ethnicities and students who felt less connected to school were more likely to engage in gambling and experience socioemotional harms.

**Conclusions:**

Our findings provide important new insights regarding risk factors in adolescence associated with gambling behaviours and socioemotional harms.

## Introduction

With growing accessibility to gambling opportunities in UK society, gambling has become a widespread and socially acceptable form of entertainment.^1^^,^^2^ Global estimates suggest that between 37.5% and 74.4% of adolescents have partaken in gambling behaviours over the past year.^3^ In the UK, generally, commercial gambling is legal only for those aged 18 and over, with two exceptions: (i) young persons between 16 and 18 can legally purchase national lottery products, including draw-based games, scratch cards and online instant wins, as well as participating in society lotteries and football pools and (ii) there are no age restrictions on Category D games machines, which include fruit machines as well as pushers and cranes.^4^ Despite this, the 2018 annual survey by the Gambling Commission^4^ found that 39% of 11–16 years old had spent their own money on a gambling activity in the 12 months prior to taking part in the study. In English adolescents, sports betting, scratch cards, instant win games and roulette are popular land-based gambling activities.^5^ Adolescents are additionally gaining access to means of gambling through the internet,^6^ with sports betting the most frequent internet-based activity for English youth.^5^

The developmental period of adolescence is characterized by increased risk-taking behaviours such as experimenting with alcohol and drugs, tobacco and unprotected sexual activity.^7^^,^^8^ Although some argue that experimentation and risk taking are essential components of healthy adolescent development,^9^ many risk-taking behaviours established in adolescence are continued into adulthood, affecting health and well-being in later life.^10^ Problem gambling defined as ‘persistent and recurrent problematic gambling behaviour leading to clinically significant impairment or distress’,^11^ affects a relatively small proportion (1.7%) of UK adolescents.^4^ It is associated with poorer psychosocial adjustment and prosocial behaviours^16^, lower self-esteem,^12^ poorer school performance^13^ and an increased risk for other addictions, delinquent behaviours and suicide.^14^ Harms associated with problem gambling can also include feelings of guilt, shame, stigma and even self-hatred,^15^ and experiencing shame has been found to contribute to problem gambling as a result of gambling to cope with negative affect.^16^ Furthermore, increased risks to problematic behaviours such as shoplifting, stealing, fighting and homelessness have been found amongst all levels of gambling behaviour, including low risk.^17^ Those who initiate gambling in adolescence are more likely to become problem gamblers in adulthood,^18^ as problem gambling is necessarily preceded by the establishment of a habitual gambling pattern.^19^ Furthermore, gambling frequency has been shown to have a dose relationship with harms.^20^

Epidemiological studies have revealed risk factors associated with problem gambling, with strong evidence that problematic gambling occurs more frequently in males,^13^^,^^14^^,^^21^^,^^22^ older adolescents,^23^ those with lower levels of education,^13^ adolescents living with parents with lower educational qualifications,^21^ ethnic minorities^24^ and children from immigrant families^25^ and those who feel less connected to school.^26^ However, relatively few studies have investigated if these risks apply to lower levels of gambling behaviours, with some exceptions (e.g.^27–29^).

Given the connection between gambling and problem gambling (and the associated harms), normalization of and accessibility to gambling opportunities, as well as increasing trajectories,^4^^,^^30^ a recent review recommended that gambling should be considered as a public health issue in Wales.^31^ From a public health perspective, it is helpful to examine the distribution of, and risk and protective factors associated with ‘all’ levels of gambling behaviours, not limited to ‘problem gamblers’.^32^ Understanding prevalence and patterns associated with lower level usage and experimentation provides an important indicator of the extent to which a behaviour is becoming normalized within society as a whole.

Thus, to address these gaps in the evidence, we draw on a large scale nationally representative survey of school children in Wales in years 7–11 to (i) describe the extent of gambling behaviours (measuring both prevalence in the last year and number and type of activities in the last week) and the socioemotional harms arising from gambling and (ii) analyze the associated demographic characteristics, school connectedness and their relationships to gambling behaviours and socioemotional harms.

## Methods

### Study population

Data were drawn from the 2017 School Health Research Network Student Health and Wellbeing (SHW) Survey, completed by students in years 7–11 from 193 secondary schools in Wales. For further details of survey please see Supplementary file. Questions about gambling were asked of about a third of all respondents based on a random route algorithm; this was done to ensure wide coverage of multiple topics while maintaining a reasonable survey length. Hence, this subsample of the SHW survey provides the analysis sample for this paper.

### Measures

We measured a count of gambling behaviours taken up in the last week using the question ‘Have you spent any of YOUR money on any of the following in the past 7 days? We want to know about games you played yourself.’ This was followed by a list of 15 possible gambling behaviours which were summed to create a count variable (see table 1).

**Table 1:** List and prevalence of gambling activities

Gambling activities	Prevalence of past 7 days gambling activities (*N*, %)
Lotto (the main national lottery draw)	838 (2.8)
National lottery scratch cards which you bought in a shop (not free scratch cards)	856 (2.9)
National lottery instant win games on the internet (e.g. national lottery gamestore)	328 (1.1)
Any other national lottery games (e.g. EuroMillions, Thunderball, Hotpicks)	311 (1.0)
Fruit machines (e.g. at an arcade, pub or club)	1376 (4.6)
Personally visiting a betting shop to play gaming machines	359 (1.2)
Playing other gambling machines	583 (2.0)
Personally placing a bet at a betting shop (e.g. on football or horse racing)	344 (1.2)
Bingo at a bingo club	545 (1.8)
Bingo somewhere other than a bingo club (e.g. social club, holiday park, etc.)	620 (2.1)
Personally visiting a casino to play casino games	229 (0.8)
Placing a private bet for money (e.g. with friends)	868 (2.9)
Playing cards for money with friends	918 (3.1)
Gambling websites/apps where you can win real money (e.g. poker, casinos, bingo, betting on sport or racing)	350 (1.2)
Other lotteries (e.g. The Health Lottery, People’s Postcode Lottery or other smaller lotteries available in shops)	225 (0.8)

All respondents in years 7–11 who answered this question (29, 807).

We also asked respondents if they had gambled in the last 12 months, without a specific definition of ‘gambling’ provided. In addition, we considered socioemotional harms arising from gambling by administering the question ‘In the past 12 months how often, if at all, would you say you have felt bad as a result of your own gambling?’ to those participants who stated they had gambled last year. Response options for this included ‘never’, ‘rarely’, ‘sometimes’, ‘often’, ‘all the time’. Responses of ‘don’t know/I do not want to answer’ were set to missing. This question was used previously in the Young Peoples Omnibus Survey.^4^ We used a set of sociodemographic characteristics as covariates, see Supplementary file for full details. Any observations where students either responded ‘I do not want to answer’ or left a question blank were set to missing.

### Analysis

All analyses were undertaken in Stata v.14.^33^ We used multiple imputation by chained equations using appropriate link functions for each variable (count, logit, ordinal logit or multinomial logit) and conditional specifications for socioemotional harms of gambling. Imputations were undertaken 20 times with 10 burn-in iterations discarded for each imputation, and were estimated separately for each grade level.

We subsequently estimated a series of single-predictor and multi-predictor regressions for each of the three gambling-related dependent variables. After estimating multi-predictor models with all covariates (described as adjusted estimates), we then tested interactions between sex and grade (described as interaction-adjusted estimates). Models for count of gambling behaviours in the last week were estimated using negative binomial regression, whereas models for any gambling in the past 12 months were estimated using logistic regression and models for socioemotional harms of gambling were estimated using ordinal logistic regression. Estimates for count of gambling activities in the last week are displayed as incidence rate ratios (IRR); i.e. they capture multiplicative differences between groups in the number of gambling activities reported (e.g. an IRR of 2 means that the group of interest reported twice as many activities as the reference group). All models included cluster-robust standard errors to account for school-level clustering.

## Results

Sample characteristics are presented in table 2. The final imputed sample included 37 363 survey respondents who were asked about gambling, which represents 35.9% of the whole sample of 103 971 respondents. Within the analysis sample, there was 18.5% missingness for gambling in the past 12 months and socioemotional harms and 20.3% for past 7 days gambling. Most covariates had low missingness (2.0% for sex, 0.0% for grade, 3.5% for ethnicity, 6.1% for family affluence, 9.2% for school belonging). Based on unimputed valid responses, the average number of gambling-related behaviours undertaken in the last week was low at 0.32, but the mean was overdispersed (SD = 1.36). The majority (59.0%) of respondents did not report any gambling in the past 12 months. The gambling activities most frequently reported were fruit/slot machines, placing a private bet for money (e.g. with friends), playing cards for money with friends, playing lotto and national lottery scratch cards (see table 1). Of those reporting any gambling in the past 12 months, 83.8% reported never feeling bad as a result of their own gambling.

**Table 2:** Sample characteristics and prevalence of gambling behaviours and socioemotional harms among 11- to 15-years old- adolescents

	Number	(%)	Number of gambling activities in the last weekMean (SD)	Number (%) participating in any gambling in the past 12 months	Number (%) who feel bad about their gambling often/all the time
**Sex**					
Male	14,011	48	0.43 (1.58)	7,094 (54)	281 (2.04)
Female	15,320	52	0.16 (0.80)	5,992 (46)	88 (0.59)
**Grade**					
Year 7	5,808	19	0.23 (1.16)	2,981 (49)	55 (0.95)
Year 8	6,328	21	0.26 (1.14)	2,850 (44)	66 (1.06)
Year 9	6,588	22	0.32 (1.35)	2,981 (43)	86 (1.33)
Year 10	5,434	18	0.35 (1.50)	2,288 (42)	101 (1.91)
Year 11	5,649	19	0.42 (1.54)	2,364 (42)	111 (2.03)
**Ethnicity**					
White British	25,063	86	0.27 (1.12)	10,893 (43)	263 (1.07)
White Other	1,266	4	0.74 (2.55)	673 (52)	46 (3.73)
Black/ethnic minority	2,733	9	0.54 (2.15)	1,355 (48)	92 (3.43)
**Family Affluence**					
Low	9,782	34	0.27 (1.25)	4,422 (44)	122 (1.28)
Moderate	9,026	32	0.28 (1.15)	4,023 (44)	115 (1.29)
High	9,625	34	0.34 (1.37)	4,125 (42)	120 (1.27)
**School belonging**					
Strongly agree	5,924	21	0.27 (1.24)	2,537 (42)	74 (1.27)
Agree	11,024	38	0.21 (.91)	4,749 (42)	76 (0.7)
Neither agree or disagree	7,226	25	0.27 (1.01)	3,120 (43)	81 (1.14)
Disagree	2,410	8	0.40 (1.55)	1,143 (47)	39 (1.69)
Strongly disagree	2,213	8	0.86 (2.76)	1,119 (50)	107 (5.02)

### Gambling activities in the last week

Regression estimates are displayed in table 3. Girls reported 62% fewer gambling activities in the last week in the fully adjusted model [adjusted IRR 0.38, 95% CI (0.35–0.42)], and older school year was associated with an increasing trajectory of count of gambling behaviours [year 11 adjusted IRR 1.65, 95% CI (1.42–1.91)], Non-White British ethnicities reported significantly greater counts of gambling behaviours [adjusted IRR 2.20, 95% CI (1.84–2.60)], but only the highest Family Affluence Scale (FAS) tertile reported a higher count of gambling behaviours [adjusted IRR 1.37, 95% CI (1.26–1.49)]. Progressively worse school belonging was also associated with greater counts of gambling behaviours [lowest school belonging adjusted IRR 3.05, 95% CI (2.58–3.60)]. The significant sex by grade interaction suggested that the age-related increase in gambling behaviours was largely restricted to boys rather than girls. For example, whereas year 11 boys reported twice as many gambling behaviours as their year 7 counterparts [interaction-adjusted IRR 2.12, 95% CI (1.79–2.49)], the interaction effect for girls [interaction-adjusted IRR 0.53, 95% CI (0.41–0.69)] suggests that compared to year 7 girls, year 11 girls have only a slight increase in count of gambling behaviours (2.12 × 0.53 = 1.12).

**Table 3:** Regression estimates of gambling behaviours and associated socioemotional harms among 11–15 years old adolescents

	How many gambling activities have you undertaken in the last week?			Have you gambled in the last year?			Of those who gambled in the last year, how often have you felt bad about your gambling?		
Covariate	IRR (95% CI)	Adj IRR (95% CI)		OR (95% CI)	Adj OR (95% CI)		OR (95% CI)	Adj OR (95% CI)	
Sex									
Male	Ref	Ref	Ref	Ref	Ref	Ref	Ref	Ref	Ref
Female	0.36 (0.33–0.41)	0.38 (0.35–0.42)	0.51 (0.41–0.63)	0.63 (0.60–0.66)	0.63 (0.60–0.66)	0.75 (0.67–0.84)	0.42 (0.37–0.47)	0.41 (0.37–0.47)	0.55 (0.41–0.73)
Grade									
Year 7	Ref	Ref	Ref	Ref	Ref	Ref	Ref	Ref	Ref
Year 8	1.11 (0.96–1.29)	1.15 (1.01–1.32)	1.19 (1.01–1.40)	0.82 (0.76–0.88)	0.79 (0.74–0.86)	0.86 (0.77–0.95)	1.23 (1.04–1.45)	1.16 (0.97–1.39)	1.25 (1.22–1.88)
Year 9	1.38 (1.20–1.59)	1.37 (1.20–1.56)	1.45 (1.25–1.70)	0.78 (0.73–0.84)	0.75 (0.70–0.81)	0.85 (0.76–0.95)	1.54 (1.30–1.83)	1.41 (1.18–1.70)	1.51 (1.22–1.88)
Year 10	1.55 (1.34–1.80)	1.34 (1.17–1.54)	1.75 (1.49–2.06)	0.75 (0.69–0.81)	0.70 (0.64–0.76)	0.78 (0.69–0.87)	1.88 (1.58–2.23)	1.68 (1.40–2.03)	1.89 (1.51–2.38)
Year 11	1.82 (1.56–2.12)	1.65 (1.42–1.91)	2.12 (1.79–2.49)	0.75 (0.69–0.82)	0.70 (0.64–0.77)	0.83 (0.73–0.94)	2.34 (1.97–2.78)	2.12 (1.77–2.54)	2.53 (2.05–3.11)
Grade by sex									
8×female	–	–	0.91 (0.70–1.20)	–	–	0.86 (0.74–1.00)	–	–	0.79 (0.56–1.11)
9×female	–	–	0.85 (0.65–1.10)	–	–	0.78 (0.66–0.92)	–	–	0.81 (0.54–1.21)
10×female	–	–	0.51 (0.38–0.67)	–	–	0.81 (0.69–0.95)	–	–	0.69 (0.46–1.03)
11×female	–	–	0.53 (0.41–0.69)	–	–	0.71 (0.60–0.85)	–	–	0.55 (0.38–0.81)
Ethnicity									
White British	Ref	Ref	Ref	Ref	Ref	Ref	Ref	Ref	Ref
White other	2.74 (2.28–3.29)	2.20 (1.84–2.63)	2.18 (1.84–2.60)	1.45 (1.29–1.62)	1.37 (1.22–1.53)	1.36 (1.22–1.52)	1.76 (1.42–2.17)	1.66 (1.33–2.06)	1.65 (1.33–2.05)
Black/ethnic minority	2.07 (1.76–2.44)	1.66 (1.45–1.89)	1.64 (1.44–1.87)	1.28 (1.18–1.39)	1.24 (1.15–1.35)	1.24 (1.14–1.34)	1.58 (1.33–1.87)	1.55 (1.31–1.83)	1.54 (1.31–1.82)
Family affluence									
Low	Ref	Ref	Ref	Ref	Ref	Ref	Ref	Ref	Ref
Moderate	1.00 (0.88–1.14)	1.09 (0.97–1.21)	1.09 (0.97–1.21)	0.97 (0.91–1.04)	0.98 (0.92–1.04)	0.98 (0.92–1.04)	1.15 (1.02–1.30)	1.18 (1.04–1.34)	1.18 (1.04–1.34)
High	1.26 (1.12–1.41)	1.37 (1.26–1.49)	1.38 (1.26–1.50)	0.91 (0.86–0.97)	0.93 (0.88–0.99)	0.93 (0.88–0.99)	1.27 (1.33–1.87)	1.31 (1.16–1.48)	1.31 (1.16–1.48)
School belonging									
Strongly agree	Ref	Ref	Ref	Ref	Ref	Ref	Ref	Ref	Ref
Agree	0.84 (0.74–0.95)	0.82 (0.74–0.92)	0.82 (0.73–0.92)	1.03 (0.96–1.10)	1.12 (1.05–1.20)	1.12 (1.05–1.20)	0.99 (0.85–1.15)	0.91 (0.78–1.07)	1.91 (0.77–1.06)
Neither agree or disagree	1.09 (0.94–1.25)	1.05 (0.92–1.20)	1.05 (0.92–1.20)	1.04 (0.97–1.11)	1.17 (1.09–1.26)	1.17 (1.09–1.26)	1.39 (1.18–1.64)	1.24 (1.04–1.47)	1.24 (1.04–1.48)
Disagree	1.64 (1.38–1.96)	1.64 (1.41–1.95)	1.66 (1.41–1.95)	1.23 (1.11–1.36)	1.41 (1.27–1.57)	1.42 (1.28–1.58)	1.44 (1.18–1.76)	1.33 (1.08–1.64)	1.34 (1.08–1.65)
Strongly disagree	3.47 (2.90–4.15)	3.05 (2.58–3.60)	3.05 (2.58–3.60)	1.40 (1.26–1.56)	1.57 (1.40–1.78)	1.58 (1.41–1.78)	2.57 (2.12–3.11)	2.27 (1.86–2.77)	2.28 (1.86–2.79)

### Gambling activities in the past 12 months

Findings for any gambling in the past 12 months were similar to findings for count of gambling behaviours for sex [adjusted IRR for girls 0.63, 95% CI (0.60–0.66)], ethnicity [Non-White British ethnicities adjusted IRR 1.37, 95% CI (1.22–1.52)] and school belonging [lowest school belonging adjusted IRR 1.57, 95% CI (1.40–1.78)]. However, increasing FAS tertile was negatively associated with gambling prevalence; this finding was significant for the highest tertile [adjusted odds ratio (OR) 0.93, 95% CI (0.88–0.99)]. Increasing school year also appeared to be protective against any past-year gambling, though age-related differences were less pronounced in adjusted models [year 11 adjusted IRR 0.70, 95% CI (0.64–0.77)].

### Socioemotional harms

Finally, findings for socioemotional harms of gambling amongst those who reported gambling in the past year also revealed unequal distribution of these harms. While girls who reported last-year gambling were less likely to feel bad about their gambling [adjusted OR 0.41, 95% CI (0.37–0.47)], increasing school year was associated with increasing socioemotional harms of gambling [year 11 adjusted OR 2.12, 95% CI (2.05–3.11)]. However, in the interaction model for socioemotional harms, as in the interaction model for count of gambling behaviours, significant interaction terms suggested that increasing harms by age were experienced more acutely by boys than by girls [interaction-adjusted IRR 0.55, 95% CI (0.38–0.81)]. Higher FAS tertiles were also associated with greater socioemotional harms [adjusted IRR 1.31, 95% CI (1.16–1.48)], as were non-White British ethnicity [adjusted IRR 1.66, 95% CI (1.33–2.06)], and worse school connectedness [adjusted IRR 2.27, 95% CI (1.86–2.79)].

### Exploratory analyses

We attempted to resolve differences between the two measures of gambling behaviour in respect of grade-related trajectories using two approaches. First, we verified findings using unimputed data, which confirmed the findings from the imputed data (findings not shown). Second, we considered the possibility that differences might be due to an increase in gambling severity by age. Put otherwise, models for count of gambling behaviours should actually consider two related data-generating processes: first, whether or not there is any gambling behaviour in the last week and second, what the count of those gambling behaviours are. We considered this hypothesis using zero-inflated negative binomial regression, which partitions and models separately these two data-generating processes (findings not shown). However, findings for the zero-inflated model yielded consistently non-significant findings for the ‘structural zero’ part of the model. That is, the hypothesis that two processes are present was unsupported.

## Discussion

This paper describes the distribution and correlates of gambling behaviours and socioemotional harms associated with gambling behaviours. Approximately two-fifths (41.0%) of respondents reported gambling in the past 12 months, this is particularly concerning given that across the UK most forms of commercial gambling is only legal for those aged 18 and over.^4^ The gambling activity most frequently reported was fruit/slot machines supporting studies of English youth.^5^ This may be particularly problematic given their availability^34^ and potential to become habitual due to high operant conditioning processes, high event frequencies, near miss opportunities and short intervals to pay out.^35^

We found significant sex differences in gambling behaviours, supporting previous findings that more males gambled in the past 12 months and gambled more frequently than females.^13^^,^^21^ Adding to the literature on social inequality highlighted^36^ we found that only the highest FAS tertile reported a higher count of gambling behaviours, similar to previous studies investigating income^37^ and comparable to studies investigating substance misuse and addiction,^41,42^ as well as greater socioemotional harms associated with gambling. Thus, students from more affluent families were more likely to have higher counts of gambling, hypothesized to be due to having more financial resources available^43^ but also feel bad about these behaviours. However, increasing family affluence appeared somewhat protective against partaking in any gambling in the last year. Non-White British ethnicities reported significantly more gambling in the last 12 months, greater counts of gambling behaviours, and socioemotional harms associated with gambling, validating previous studies^44^ and contributing to recommendations to pay greater attention to ethnic differences in the study of adolescent gambling.^14^ Additionally, we found that students who feel less connected to school were more likely to engage in greater counts of gambling behaviours and experience socioemotional harms arising from gambling, expanding previous findings investigating school connectedness and problem gambling^26^ to any gambling activities, and similar to studies showing that students who gamble more frequently typically have worse school performance^45^ which could be indicators of not feeling connected to school. This is consistent with studies investigating numerous other risk behaviours.^46^

Older school year was associated with an increasing trajectory of count of gambling behaviours (replicating previous findings^23^). The significant sex by grade interaction suggested that the increase in gambling behaviours was largely restricted to boys rather than girls. In addition, older school year was associated with increasing socioemotional harms of gambling, with interaction terms suggesting that harms by age were experienced more acutely by boys than by girls. This could be linked to establishing masculine identities as certain forms of gambling perceived to being risky, or skill-based, are associated with higher levels of conformity to masculine norms.^47^

The contradictory findings for the relationship between grade and gambling behaviours merit some discussion, especially as these contradictory findings were also mirrored in the relationship between FAS and gambling. Whilst the majority of studies on adolescent problem gambling have found age-related progression, our finding is similar to other studies investigating lower levels of gambling, e.g.^48^ found little variability in rates of any gambling measured during the previous year. Despite this we explored several possible reasons for these contradictions, including issues in the imputation model or the presence of two separate data-generating processes, but neither of these explanations were supported. Thus, the most likely explanation relates to the measurement methods used to capture gambling behaviours. It is possible that the count-based variable may be more effective as to measure gambling this did not presume a specific shared definition of what gambling is. In contrast, the question about last-year gambling was presented without definition or description, and increasing age could be related to changing understandings of what ‘counts’ as gambling. This may have limited the validity of this question in capturing the ‘fact’ of gambling behaviours, a problem to which the gambling count variable was less susceptible. However, the gambling count variable relied on a list of 15 specific gambling behaviours (e.g. national lottery scratch cards, fruit machines, visiting a betting shop) and may have missed culturally or regionally salient forms of gambling that might be predictive of future disorder and harms.

### Strengths and limitations

In the largest analysis of its kind in a UK sample, we described the distribution and correlates of gambling behaviours and socioemotional harms associated with gambling behaviours. Using a public health approach, this paper offers a broader viewpoint on gambling, not restricted to problem gambling. However, the present study is not without its limitations. The data were cross-sectional, thus, causal inferences cannot be drawn. No validated measures of problem gambling were used; instead we used existing questions from the Gambling Commission ‘Young People and Gambling’ Survey 2017.^49^ This may be particularly problematic for the measurement of socioemotional harms, which used a single item, not been confirmed to be a valid measure of harm. However, the main emphasis of this study was to examine gambling behaviours not problem gambling. Whilst multiple imputation was used to account for missing data, the relatively high amount of missing values related to the three outcome measures should be noted. In addition, self-reported data may have been biased by standard limitations (e.g. memory recall biases, social desirability, etc.), particularly considering the sensitive nature of certain survey questions.

### Implications for intervention and research

Given the widespread opportunity to gamble and lack of education regarding its associated risks,^30^ adolescents are vulnerable to poor outcomes associated with gambling. The finding that increasing school year was associated with increasing socioemotional harms of gambling, and harms were experienced more acutely by boys adds to the literature and may be important for designing and evaluating interventions. Whilst specific programs may be effective,^50,51^ research has shown that adolescent problem gamblers are reluctant to seek help,^50^ usually experiencing significant difficulties before treatment is sought.^2^ Thus, these findings support previous policy recommendations to work with schools and the education sector to provide awareness materials on gambling harms for students and their parents, complementing the inclusion of content on gambling harms, resilience and well-being in the All Wales Schools Liaison Core Programme.^52^ Findings support the Chief Medical Officer for Wales’ recommendation that parents, guardians, and those responsible for the health and well-being of children and vulnerable people should be aware of the harms, and potential harms, of gambling to mitigate harm.^31^ Furthermore, the prevalence of young people gambling (despite current UK age restrictions) supports a call to action for cultivating protective factors such as parent supervision.^38^

Prevention approaches across a number of levels^46^ have been shown to be effective in preventing other risk-taking activities such as adolescent substance misuse.^53^ Given our findings that students who feel less connected to school were more likely to engage in gambling and experience socioemotional harms, school environment interventions such as INCLUSIVE^54^ and SEHER^55^ which support pupils’ commitment to their school community as a means of removing the need for young people to engage in alternative markers of identity and status through risky behaviours such as violence and substance use may offer promise for reducing gambling.

### Conclusion and suggestions for future research

Our findings provide important new insights regarding the distribution and determinants of experimentation with gambling in adolescence. Given that a greater proportion of year 7’s had gambled in the last year compared to older grades, further research investigating type of gambling behaviour and age may be worthwhile. Further research into the links between specific gambling behaviours (e.g. number and type of gambling activities) and later gambling problems are needed. Future studies should consider how adolescents specifically view gambling-like behaviours within online gaming and whether this predicts future engagement in gambling. Longitudinal analyses would allow investigation of factors that are predictive of future problem gambling including adulthood,^56^ e.g. the causal role of income inequality.^36^ This would provide a broader perspective in line with the public health approach to tackling gambling-related harm and inform policy development in this area.

## Supplementary data

Supplementary data are available at *EURPUB* online.
