# Corrigendum

**DOI:** 10.1093/eurpub/ckz227

**Published:** 2020-07-20

**Prevalence of gambling behaviours and their associations with socioemotional harm among 11–16 year olds in Wales: findings from the School Health Research Network survey** G.J. Melendez-Torres, Rebecca E. Anthony, Gillian Hewitt, Simon Murphy, Graham F. Moore.

There were several transcription and tabulation errors in Table 2 in the original paper. These have now been corrected and the corrected table appears below.

**Table 2:** Sample characteristics and prevalence of gambling behaviours and socioemotional harms among 11- to 15-years old- adolescents

	Number	(%)	Number of gambling activities in the last week Mean (SD)	Number (%) participating in any gambling in the past 12 months	Number (%) who feel bad about their gambling often/all the time
**Sex**					
Male	14,011	48	.43 (1.58)	7,094 (54)	281 (2.04)
Female	15,320	52	.16 (0.80)	5,992 (46)	88 (0.59)
**Grade**					
Year 7	5,808	19	.23 (1.16)	2,981 (49)	55 (0.95)
Year 8	6,328	21	.26 (1.14)	2,850 (44)	66 (1.06)
Year 9	6,588	22	.32 (1.35)	2,981 (43)	86 (1.33)
Year 10	5,434	18	.35 (1.50)	2,288 (42)	101 (1.91)
Year 11	5,649	19	.42 (1.54)	2,364 (42)	111 (2.03)
**Ethnicity**					
White British	25,063	86	.27 (1.12)	10,893 (43)	263 (1.07)
White Other	1,266	4	.74 (2.55)	673 (52)	46 (3.73)
Black/ethnic minority	2,733	9	.54 (2.15)	1,355 (48)	92 (3.43)
**Family Affluence**					
Low	9,782	34	.27 (1.25)	4,422 (44)	122 (1.28)
Moderate	9,026	32	.28 (1.15)	4,023 (44)	115 (1.29)
High	9,625	34	.34 (1.37)	4,125 (42)	120 (1.27)
**School belonging**					
Strongly agree	5,924	21	.27 (1.24)	2,537 (42)	74 (1.27)
Agree	11,024	38	.21 (.91)	4,749 (42)	76 (0.7)
Neither agree or disagree	7,226	25	.27 (1.01)	3,120 (43)	81 (1.14)
Disagree	2,410	8	.40 (1.55)	1,143 (47)	39 (1.69)
Strongly disagree	2,213	8	.86 (2.76)	1,119 (50)	107 (5.02)

